# Evaluation of a Rapid Urine Antigen Detection Assay as a Point-of-Care Test in the Diagnosis of Community-Acquired Pneumonia

**DOI:** 10.7759/cureus.44078

**Published:** 2023-08-24

**Authors:** Mohammed Khaleel, Sara Samreen, Saritha Sirangi, Mummareddi Dinesh Eshwar, Padmaja R. M., Kalyani Dhanekula

**Affiliations:** 1 Microbiology, Mahavir Institute of Medical Sciences, Vikarabad, IND; 2 General Surgery, Deccan College of Medical Sciences, Hyderabad, IND; 3 Internal Medicine, Mahavir Institute of Medical Sciences, Vikarabad, IND

**Keywords:** pneumonia, point-of-care test, urine, rapid antigen test, streptococcus pneumoniae, community-acquired pneumonia (cap)

## Abstract

Introduction

Community-acquired pneumonia (CAP) is among the most common public health problems encountered throughout the world. CAP is a frequent cause of lower respiratory tract infections among children and geriatric-age persons. The etiology of CAP is complex but generally involves infection with bacteria like *Streptococcus* *pneumoniae *(*S*. *pneumoniae*), which is the most common cause of CAP. The underdiagnosis of CAP due to the limitations of conventional culture methods could be responsible for severe morbidity and mortality, especially among susceptible populations. We evaluated the usefulness of a rapid immunochromatographic test (BinaxNOW™, Abbott, Chicago, IL) that detects *S*. *pneumoniae *through a rapid urine antigen test (RUAT) as a point-of-care (POC) diagnostic method in the early detection of CAP.

Methods

A prospective study was conducted in a university-affiliated teaching hospital between January 2019 and September 2019 (nine months). The study recruited 300 inpatients who revealed signs and symptoms associated with pneumonia. The study was approved by the institutional ethics committee, and all participants provided their voluntary informed consent. Laboratory evaluation included the collection of sputum samples, which were processed for Gram stain and routine culture. Five milliliters of blood were collected from all the subjects for carrying out a blood culture. A urine sample was collected from each participant for the detection of *S*. *pneumoniae *through the point-of-care urinary antigen test.

Results

Of the 300 patients diagnosed with CAP, the *S*. *pneumoniae* RUAT was positive in 110 out of 140 cases of pneumococcal pneumoniae (78.57%). The RUAT results were positive for 20 (66.6%) out of 30 bacteremic patients and for 90 (81.8%) out of 110 patients positive for sputum culture. The RUAT was positive in 10 out of 20 cases of pneumonia with an unknown microbial etiology. The overall sensitivity (78.57%), specificity (100%), positive predictive value (100%), negative predictive value (98.88%), and accuracy (90%) of the RUAT were similar to sputum culture results.

Conclusion

The RUAT has shown comparable efficacy with sputum culture and therefore can be used as a complementary approach to conventional methods in the early diagnosis of CAP caused by *S*. *pneumoniae. *Due to its ease of use and rapid results, it could be incorporated as a POC diagnostic test.

## Introduction

Community-acquired pneumonia (CAP) is the second most common cause of mortality in India. Children below five years of age and older adults were found to be increasingly susceptible to CAP. The predisposing factors for CAP in persons of extreme age groups include lowered immunity and the presence of underlying chronic conditions [1]. Other risk factors include asthma, chronic obstructive pulmonary disease (COPD), chronic lung disease, smoking, diabetes mellitus, alcoholism, and chronic heart and liver disease [2].

​​​​​​*Streptococcus pneumoniae *(*S*. *pneumoniae*) is commonly present as an indigenous flora of the oropharynx in healthy individuals. However, the carrier rate varies according to age, environment, season, and the presence of an upper respiratory infection. *S*. *pneumoniae *is the leading cause of community-acquired pneumonia (CAP) and is also a major opportunistic pathogen in healthcare-associated pneumonia (HCAP) [3].

Currently, the incidence of CAP is on the rise, and the challenges of diagnosis remain a cause for serious concern among clinicians. Additionally, patients with CAP often present late with their symptoms, thereby causing delays in the timely diagnosis. Moreover, the use of broad-spectrum antibiotics has led to an increase in resistance among bacteria, which culminates in treatment failures [4].

The bacteria most commonly associated with CAP is *S*. *pneumoniae*. Other agents that can be responsible for CAP include *Streptococcus pyogenes*, *Haemophilus influenzae*, *Klebsiella pneumoniae*, *Pseudomonas aeruginosa*, and others [5,6]. 

Infection with *S*. *pneumoniae *remains underdiagnosed due to the limitations of the routinely used conventional methods. The blood culture, along with sputum gram staining and its culture, is regarded as the gold standard in the diagnosis of CAP [7]. However, these methods have several drawbacks that contribute to difficulties in diagnosis and patient management.

The need of the hour is a point-of-care (POC) test for the detection of the etiological agent of CAP that allows clinicians to choose initial appropriate antimicrobial therapy [8]. The POC testing is easy to do and performed on the bedside of the patients, which minimizes the time for laboratory identification of the etiological agents and enables physicians to better manage patients, thereby reducing morbidity and mortality. We, therefore, attempted to evaluate the utility of a rapid urinary antigen test (RUAT) that detects *S*. *pneumoniae *C-polysaccharide (a cell wall component) in the diagnosis of CAP.

## Materials and methods

A prospective study was conducted in a university-affiliated teaching hospital between January 2019 and September 2019 (nine months). The study was approved by the Institutional Ethics Board (IRB) of the Deccan College of Medical Sciences and Allied Hospitals, Hyderabad (Ref. No. 2019/26/008). A total of 300 patients who were provisionally diagnosed as suffering from CAP based on clinical signs and symptoms were included in the study.

The patient's data collected included the presence of comorbidities, antibiotic therapy prior to admission to the hospital, clinical signs, and radiological features. Comorbidity was described as the presence of any diseases for which the patient was under constant medical management or was obtaining any therapy, including pulmonary diseases like asthma and COPD and other diseases including ischemic heart disease, cardiac failure, diabetes mellitus, and cerebrovascular disease.

Inclusion and exclusion criteria

All patients diagnosed with CAP based on clinical features and chest radiographs performed at the time of admission consistent with pneumonia and persons without any pre-existing illnesses were included in the study. Patients aged above 60 years and presenting with clinical features compatible with an acute lower respiratory tract infection wherein the patients develop a fever (body temperature >37.8°C), perspiration, body aches, headaches, nasopharyngitis, cough with sputum production, pleuritic chest pain, shortness of breath, and pulmonary consolidation were included in the study.

All patients who were hospitalized within the last 15 days with pneumonia and/or bronchial obstruction, patients with lung cancer, patients suffering from bronchoaspiration, and patients who lost their follow-up were excluded from the study.

Microbiological examinations

A sputum sample for gram stain and culture, blood for culture, and a urine specimen for the detection of S. pneumoniae antigen were obtained from each study subject. The quality of sputum samples included were those that showed the presence of >25 white blood cells or polymorphonuclear leukocytes, and <10 squamous epithelial cells per low-power (10×) magnification field. Conventional and standard microbiological techniques were employed to carry out the identification of the bacterium. 

The POC rapid urine antigen test

**Figure 1:**
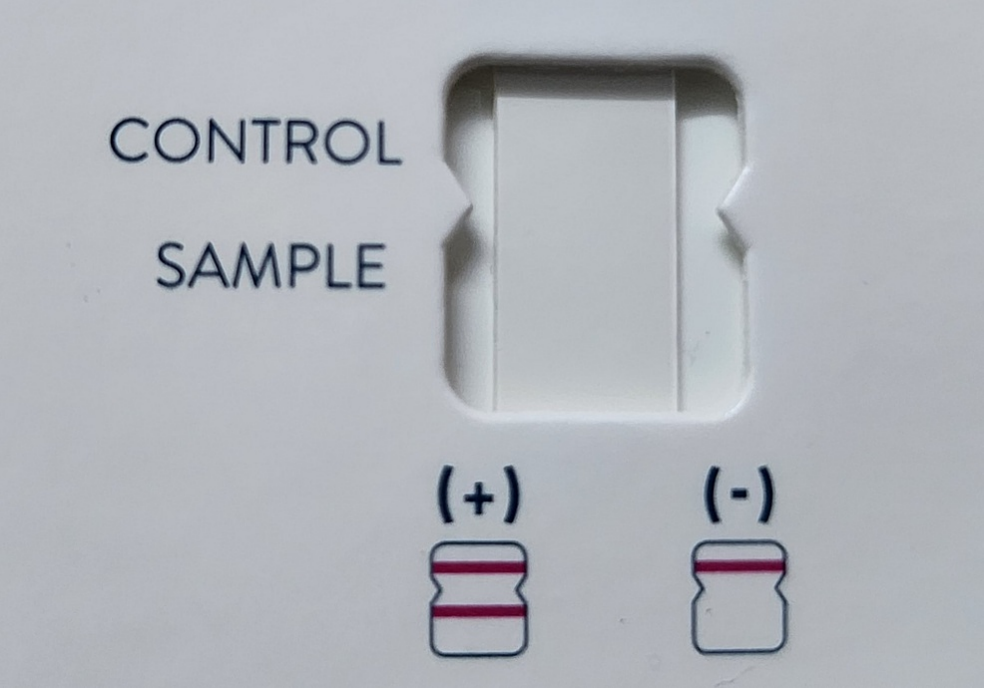
Interpretation of rapid urine antigen test result. +: Positive; –: Negative.

The commercially available rapid urine antigen test (RUAT) (BinaxNOW™, Abbott, Chicago, IL) for *S*. *pneumoniae *works as an immunochromatographic test (ICT). This is a rapid test used for the qualitative detection of *S*. *pneumoniae *urinary antigen, wherein the results appear within 15 minutes. A sterile cotton swab was immersed into the urine sample and placed on a nitrocellulose membrane that had previously been impregnated with complexes of rabbit antibodies that were conjugated with colloidal gold particles against the *S. pneumoniae* antigen. If the patient sample consists of the antigen, the conjugate combines with it to produce a positive reaction. The test result was interpreted by the presence or absence of visually detectable pink-to-purple-colored bands in the test and control areas, as shown in Figure 1.

Statistical analysis

The collected data were systematically entered into the Microsoft Excel sheets (Microsoft Corporation, Redmond, Washington, USA) and analyzed using SPSS Statistics for Windows, Version 17.0, 2008 (SPSS Inc., Chicago, Illinois).

## Results

Of the total 300 patients included in the study, 210 (70%) were male and 90 (30%) were female. All the patients included in the study were aged above 60 years. A significant number (120) of patients were those who quit smoking (40%). Chronic obstructive pulmonary disease (COPD) (43.44%) was the most common comorbidity noted among the study participants, followed by diabetes mellitus (40%), asthma (36.66%), and ischemic heart disease (33.33%). The details of the patient characteristics can be depicted in Table 1.

**Table 1:** Patient characteristic features. COPD: chronic obstructive pulmonary disease; ICU: intensive care unit.

Parameter	Variable	Number n (%)
Sex	Male	210 (70)
	Female	90 (30)
Age (years)	>60	300 (100)
Smoking habits	Current cigarette smokers	80 (26.66)
	Quit smoking	120 (40)
	Non-smoker	100 (33.33)
Comorbidity	COPD	130 (43.33)
	Asthma	110 (36.66)
	Ischemic heart disease	100 (33.33)
	Diabetes mellitus	120 (40)
	Cerebrovascular disease	20 (6.66)
	Prior antibiotic therapy	80 (26.66)
	ICU admission	40 (13.33)

The microbiological culture results revealed *S*. *pneumoniae *in 140 patients followed by *Staphylococcus aureus *in 80 patients, *Haemophilus influenzae *in 60 patients, and 20 patients showed negative results on culture. Out of 140 cases of *S*. *pneumoniae*, 110 were detected by sputum culture, and 30 were detected by blood culture.

When the 140 patients who were diagnosed based on culture were compared with the rapid urine antigen test, only 110 out of the 140 patients (78.57%) demonstrated a positive result. Conversely, the rapid urine antigen test results were positive for 20 (66.6%) out of 30 bacteremic patients and 90 (81.8%) out of 110 patients who were positive for sputum culture. The urinary antigen test was positive in 10 (50%) of 20 cases of unknown microbial etiology on culture. The details of the etiology based on the different tests performed are detailed in Table 2.

**Table 2:** Etiological agents and positivity in relation to the diagnostic test.

Etiological agent (total positives)	Sputum culture	Blood culture	The rapid urine antigen test
*Streptococcus pneumoniae* (140)	110	30	110
*Staphylococcus aureus* (80)	80	00	00
*Haemophilus influenzae* (60)	60	00	00
Unknown etiology (20)	00	00	10

Among the 110 RUAT positives, only 90 were sputum culture positive. Conversely, 20 samples that were sputum culture positive revealed negative RUAT. 20 samples revealed positive RUAT out of the 30 positive blood cultures. The overall sensitivity, specificity, positive predictive value (true positive), negative predictive value (true negative), and accuracy of the RUAT, sputum culture, and blood culture are detailed in Table 3.

**Table 3:** Diagnostic efficacy of tests.

Diagnostic test	Sensitivity (%)	Specificity (%)	Positive predictive value (%)	Negative predictive value (%)	Accuracy (%)
The rapid urine antigen test	78.57	100	100	98.88	90
Sputum culture	78.57	100	100	98.88	90
Blood culture	21.43	100	100	96.03	63.33

## Discussion

*Streptococcus* *pneumoniae *is the most common cause of CAP, and many cases of pneumonia caused by this bacterium remain underdiagnosed due to the limitations of conventional laboratory methods used for the diagnosis [8,9]. The recently developed BinaxNOW™ *S*. *pneumoniae *RUAT has caught the interest of many clinicians [10]. This was due to the fact that RUAT had eased the diagnosis of CAP caused by *S*. *pneumoniae*.

In this study, we evaluated the performance of BinaxNOW™ *S*. *pneumoniae *RUAT when compared to culture (sputum and blood), which is considered the gold standard. Good quality sputum is difficult to obtain in >50% of patients, and it is prone to misdiagnosis due to the presence of normal microflora. Additionally, other invasive samples like bronchoalveolar lavage are not accomplished routinely. Although the results of the blood culture in this study revealed 100% specificity, they demonstrated low sensitivity (21.43%).

The RUAT demonstrated similar sensitivity (78.57%), high specificity (100), and positive predictive value (98.88%) as the sputum culture. These findings are similar to studies of Ercis et al., in which the sensitivity and specificity of RUAT were shown to be 72.7% and 97.6%, respectively [11]. The improved sensitivity of BinaxNOW™ *S. pneumoniae* RUAT could be due to the detection of capsular polysaccharide, which is common to all strains.

The RUAT was performed in unconcentrated urine. Unconcentrated urine samples require less time to be processed (about half an hour), allowing them to be used as bedside diagnostic tools. The unconcentrated urine was RUAT negative but blood culture positive in four cases in a previous study from New Zealand [12]. However, there is no conclusive evidence that shows the necessity of the use of concentrated urine to improve the yield of RUAT.

The RUAT had an additional advantage, as evidenced by the results, wherein we detected two additional positive cases in comparison with sputum culture results. Hence, the utility of sputum culture alone in the diagnosis of CAP appears to miss some cases. On the other hand, the sheer isolation of *S*. *pneumoniae *in sputum culture does not necessarily confirm the diagnosis. This could be due to the fact that this bacterium can colonize the lower respiratory tract of healthy individuals [13].

The blood culture alone showed less sensitivity and therefore appears to be ill-suited for the diagnosis of CAP [14]. This could be due to the fact that patients could have used antibiotics prior to presenting to the hospital. Moreover, a higher bacterial load in the blood is required to improve the chances of isolation. 

Our study was limited to adult patients since previous studies that assessed the efficacy of RUAT in children found it unsuitable for detecting pneumococcal pneumonia in those who had nasopharyngeal colonization [15]. It, therefore, confirms that sputum culture may return false-positive results owing to the presence of normal flora.

Since RUAT has shown comparable efficacy to the culture of sputum, it can lead to an early diagnosis of CAP. The RUAT results may be utilized to initiate empirical antimicrobial therapy during patient management and minimize the delay due to culture. Additionally, the application of RUAT as a POC may contribute to decreasing resistance to antibiotics and also reduce the treatment cost.

Study limitations

This work was carried out as a pilot study for nine months and included a very limited number of patients. Therefore, further large-scale studies involving a higher number of patients are required to replicate the results obtained in this study. Additionally, this study was carried out only among patients aged above 60 years. 

## Conclusions

The results of this study show that *S*. *pneumoniae *is the most common etiological agent of CAP. The RUAT has revealed comparable efficacy to sputum culture. Blood culture showed the least sensitivity and low accuracy in the diagnosis of CAP. The RUAT could be used as a POC diagnostic test that may contribute to the early diagnosis of CAP. The RUAT results may be applied among adult patients with comorbidities to initiate empirical treatment and minimize morbidity and mortality. Moreover, RUAT results are not influenced by prior antibiotic therapy. 
